# Optimizing Muscle Performance in Young Soccer Players: Exploring the Impact of Resisted Sprint Training and Its Relationship with Distance Covered

**DOI:** 10.3390/sports13010026

**Published:** 2025-01-20

**Authors:** Felipe Hermosilla-Palma, Juan Francisco Loro-Ferrer, Pablo Merino-Muñoz, Nicolás Gómez-Álvarez, Rodrigo Zacca, Hugo Cerda-Kohler, Ciro Brito, Jorge Pérez-Contreras, Moacyr Portes-Junior, Esteban Aedo-Muñoz

**Affiliations:** 1 Escuela de Doctorado, Universidad de Las Palmas de Gran Canaria, 35016 Las Palmas de Gran Canaria, Spain; 2 Pedagogía en Educación Física, Facultad de Educación, Universidad Autónoma de Chile, Talca 3460000, Chile; 3 Núcleo de Investigación en Ciencias de la Motricidad Humana, Universidad Adventista de Chile, Camino a Tanilvoro Km 12, Chillán 3780000, Chile; pablo.merino@usach.cl (P.M.-M.); nicolasgomez@unach.cl (N.G.-Á.); 4 Departamento Ciencias Clínicas, Universidad de Las Palmas de Gran Canaria, 35016 Las Palmas de Gran Canaria, Spain; juanfrancisco.loro@ulpgc.es; 5 Programa de Engenharia Biomédica, Instituto Alberto Luiz Coimbra de Pós-Graduação e Pesquisa de Engenharia (COPPE), Universidade Federal do Rio de Janeiro, Rio de Janeiro 21941-853, Brazil; 6 Research Center in Physical Activity, Health and Leisure (CIAFEL), Faculty of Sports, University of Porto (FADEUP), 4200-450 Porto, Portugal; rzacca@fade.up.pt; 7 Laboratory for Integrative and Translational Research in Population Health (ITR), 4050-600 Porto, Portugal; 8 Departamento de Educación Física, Deportes y Recreación, Facultad de Artes y Educación Física, Universidad Metropolitana de Ciencias de la Educación, Santiago 7760197, Chile; hugorck@gmail.com; 9 Laboratory of Psychophysiology and Performance in Sports and Combats, Postgraduate Program in Physical Education, School of Physical Education and Sport, Federal University of Rio de Janeiro, Rio de Janeiro 21941-853, Brazil; 10 Department of Physical Education, Federal University of Juiz de Fora, Governador Valadares 35010-180, Brazil; ciro.brito@usach.cl; 11 Escuela de Ciencias de la Actividad Física, El Deporte y la Salud, Facultad de Ciencias Médicas, Universidad de Santiago de Chile, Santiago 8370003, Chile; esteban.aedo@usach.cl; 12 Escuela de Ciencias del Deporte y Actividad Física, Facultad de Salud, Universidad Santo Tomas, Santiago 8370003, Chile; 13 Escuela de Educación, Magíster en Evaluación y Planificación del Entrenamiento Deportivo, Universidad Viña del Mar, Viña del Mar 2572007, Chile; 14 Laboratorio de Biomecánica Deportiva, Unidad de Ciencias Aplicadas al Deporte, Instituto Nacional de Deportes, Santiago 7780421, Chile

**Keywords:** resistance training, muscle strength, football, physical fitness, puberty players

## Abstract

Background: Speed training with resisted sprints has been shown to positively affect neuromuscular performance in soccer players. Various loads, ranging from 10% to 120% of body mass, have demonstrated performance improvements across the spectrum. However, the impact of sprint distance with optimal load on these adaptive responses has yet to be thoroughly described. Objective. To analyze the influence of sprint distance in resisted sprints on muscle performance in young soccer players. Methods. This quantitative study utilized a pre-post experimental design. The sample consisted of 24 young soccer players (15.3 ± 0.68 years; 61.4 ± 7.08 kg; 1.60 ± 0.06 m) randomized into three groups (10, 20, and 30 m) and subjected to 12 sessions of resisted sprint training over six weeks. The volume was homogenized across groups, with a total distance of 120 m for each. The intervention’s effect was analyzed through performance in the isometric mid-thigh pull (IMTP), countermovement jump (CMJ), modified 505 agility test (505 m), and linear sprint tests. Differences were analyzed using a mixed ANOVA, incorporating a between-subjects factor (training group) and a within-subjects factor (pre- and post-intervention). Results. Time-dependent differences were observed in all groups for peak force (PF) (*p* < 0.001; η^2^p = 0.62), time to PF (TPF) (*p* < 0.001; η^2^p = 0.53), impulse at 50 (*p* < 0.001; η^2^p = 0.57), 100 (*p* < 0.001; η^2^p = 0.60), and 200 ms (*p* < 0.001; η^2^p = 0.67) in IMTP; jump height by impulse-momentum (*p* < 0.001; η^2^p = 0.64), rate of force development (*p* = 0.04; η^2^p = 0.14), yielding impulse (*p* < 0.001; η^2^p = 0.49), and concentric impulse (*p* = 0.01; η^2^p = 0.19) in CMJ; time (*p* < 0.001; η^2^p = 0.46) in 505 m; and average speed in linear sprint (*p* = 0.003; η^2^p = 0.36), with moderate to large effect sizes, regardless of the distance covered. No differences were observed for the interaction between the time* and group or between groups. Conclusion. Performance improvements were independent of the sprint distance, with no differences between training groups. Distances between 10 and 30 m may enhance muscle performance in young soccer players.

## 1. Introduction

Soccer alternates between high- and low-intensity actions, where strength and power expression are key factors in high-intensity player performance. High-intensity actions, such as sprints, accelerations, decelerations, and changes of direction (COD) [1,2,3,4], play a key role in goal-related situations, with linear sprinting being decisive in over 60% of assists and goals scored by players [5]. Most sprints in soccer occur over short distances (0 to 30 m) [6,7], making acceleration development crucial. Regarding COD, these actions are among the most prevalent in a match, with approximately 700 efforts per game [1]. Accordingly, COD is essential for evading opponents and gaining advantageous positions. Additionally, COD is an implicit skill in agility, including acceleration, deceleration, and decision-making situations [8], highlighting its importance for performance development.

Concerning the methods to develop this quality, they can be divided into three subgroups according to task specificity [9]: (a) Primary methods, which are based on sprint-specific actions; (b) Secondary methods, which also focus on sprint patterns but incorporate overload or underload conditions; and (c) Tertiary methods, characterized by the inclusion of non-sprint-specific actions, such as plyometrics or resistance training. Speed training with resisted running (secondary methods) has proven effective for soccer players of different ages and genders [10,11,12]. Various devices and force vectors have been experimented with in this context [13], with sled running being an effective method for increasing acceleration capacity [14,15]. This type of intervention also reports improvements in a wide range of related actions, such as changes of direction, jump height (SJ-CMJ), average power, and propulsive average power [11,16,17]. It is important to highlight that these capacities undergo accelerated development in tandem with the processes of biological maturation [3]. Moreover, manipulating the external training load’s variables is crucial for achieving performance improvements [18].

The dosing of resisted sprint loads ranges from 10% to 120% of body mass. Performance improvements have been reported across the spectrum [17,19,20,21]. These studies have shown that this dosing increases maximal power and speed over 5 and 30 m. It also optimizes the horizontal force-velocity profile. Theoretically, training with a resisted-sled load that induces a ~50% decrement in maximum velocity (i.e., optimal load) increases the ability to produce maximal power output. It also leads to a practical increase in the ability to transfer force throughout the sprinting phases. This results in an increase in both force and velocity capacities [22]. Runs of up to 30 m are key for promoting acceleration development, as this phase predominantly occurs within that distance [23]. Finally, factors such as the athletes’ training status and the manipulation of the training load can influence the achievement of optimal adaptations [14].

Heavy loads, ranging from 75–112% of body weight (BW), improve the expression of the mechanical components of sprinting (force, velocity, and maximal power). These results persist residually, lasting even after four weeks without specific intervention [17]. However, there is evidence pointing to the detrimental effect of loads that reduce maximal sprint speed by more than 30%, as they can negatively impact running technique [9]. This is an important consideration for physical conditioning coaches. Similarly, both heavy and light loads enhance performance in early and late acceleration phases [24]. These improvements are evident in within-group comparisons. However, their effectiveness over sprint training without resistance remains inconclusive [25,26].

Regarding the effects on other explosive actions, it has been shown that resisted sprints with 30% body weight loads do not improve change-of-direction performance, nor vertical jump height [27]. It has been proposed that the inclusion of combined strength stimuli in both the vertical and horizontal planes would be more suitable for improving this expression of performance [28]. Methodological guidelines suggest that programming based on velocity loss percentages may be more suitable for improving acceleration [24].

Despite these findings, no studies have incorporated the manipulation of sprint distance as an independent variable to verify its influence on neuromuscular performance improvements. Therefore, the present study aims to analyze the influence of different resisted sprint distances on neuromuscular performance in young soccer players. The research hypothesis proposes that certain distances maximize muscular adaptations during high-intensity efforts.

## 2. Materials and Methods

### 2.1. Experimental Approach to the Problem

**Figure 1:**
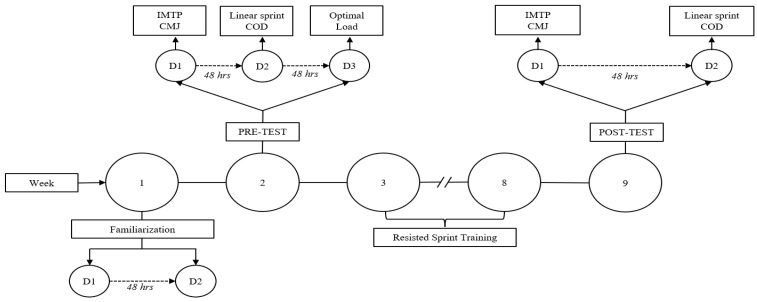
Experimental design. Temporal sequence of resisted sprint training protocol. Total volume: nine weeks (Week 1 = familiarization; Week 2 and 9 = pre- and post-test, respectively; Week 3 to 8 = resisted sprint training). IMTP: Isometric mid-thigh pull; CMJ: Countermovement jump; COD: Change of direction; D: Day.

The study was conducted with young male soccer players from a professional Chilean club. The training program was executed over nine weeks (see Figure 1) between March and June during the competitive season. Week 1 was dedicated to familiarization, during which two sessions of sled drags with loads of 10% and 20% of body mass were performed. During this period, mass and height were evaluated using a scale (SECA model 803) and a stadiometer (SECA model 213) with an accuracy of 0.1 kg and 0.1 m, respectively. Subsequently, assessments were conducted before and after (weeks two and nine) the 12 sessions of resisted sprint training. The evaluations were performed over two days, with 48 h between them: Day 1 included (a) measurements of height and mass, (b) an isometric mid-thigh pull, and (c) a countermovement vertical jump; Day 2 included (a) a 30 m linear sprint and (b) a change of direction (m505). After the initial assessments, subjects were randomized into three groups, each with different sprint distances: G10m (10 m—n = 8), G20m (20 m—n = 7), and G30m (30 m—n = 9), using Excel’s RAND() function for random assignment. Between weeks 3 and 8, 12 sessions of resisted sprints with differential distances and optimal load were applied. The optimal load is defined as reducing maximum speed by 50%. This load magnitude maximizes horizontal power production [29]. The sessions were supervised by the study’s principal investigator and the team coaches. All procedures were conducted at the club’s outdoor facilities, with a temperature range of 17° to 20 °C.

### 2.2. Participants

Twenty-four young male soccer players (15.9 ± 0.69 years; 61.4 ± 7.08 kg; 1.69 ± 0.06 m) from the S15 and S16 categories of a professional Chilean soccer club voluntarily participated in the study. Participants were selected through convenience sampling. All players had a weekly training frequency of six sessions, including the official competition. At the start of the intervention, they were informed about the benefits and potential risks of the research. They expressed their willingness to participate by signing informed assent and consent forms, signed by their parents or legal guardians. The study was conducted according to the ethical standards for research involving humans, as stated in the Declaration of Helsinki. It was approved by the Ethics Committee of Adventist University of Chile, Chillán, Chile (resolution 2023-07, Acta No. 2023-04, and vote No. 2023-08).

### 2.3. Procedures

#### 2.3.1. Performance Tests

##### Isometric Mid-Thigh Pull (IMTP)

The IMTP test was conducted on day one of weeks two and nine (see Figure 1). The procedure for the test execution aligns with previously described methods [30]. Specific activation included three attempts of IMTP at 50%, 75%, and 90% of perceived effort, with a one-minute rest between them. Following this, three maximal attempts were performed with the instruction, “Push your feet into the ground as quickly and forcefully as possible.” Each attempt lasted eight seconds, with the first three seconds for preparation and the remaining five seconds for effort. The rest period between attempts was two minutes. Data were recorded using force plates (PASPORT force plate, PS-2141, PASCO Scientific, Roseville, CA, USA) via SPARKvue software (version 4.6.1, Roseville, CA, USA), then exported to an Excel spreadsheet (version 16, Microsoft, Redmond, WA, USA) and finally processed in Matlab (version 9.6, Natick, MA, USA). The onset of both tests was estimated using a five-standard-deviation change in the force-time curve [31]. Analyzed variables included absolute peak force (IPF) and impulse at 50, 100, and 200 ms.

##### Countermovement Vertical Jump (CMJ)

The CMJ test was conducted on day one of weeks two and nine (see Figure 1). After a specific activation of five submaximal jumps, players performed three maximal CMJ attempts with a two-minute rest between them. The depth of the descent was self-selected for comfort. Players were instructed to jump “as quickly and as high as possible” [32]. The jumps were performed with hands fixed on the hips. Data were recorded using force plates (PASPORT force plate, PS-2141, PASCO Scientific, Roseville, CA, USA) via SPARKvue software (version 4.6.1, Roseville, CA, USA), then exported to an Excel spreadsheet (version 16, Microsoft, Redmond, WA, USA) and finally processed in Matlab (version 9.6, Natick, MA, USA). Analyzed variables included jump height (JH) calculated through impulse momentum, rate of power development (RDP), yielding impulse (IY), braking impulse (IB), and concentric impulse (IC).

##### Linear Sprint

Speed tests were conducted on day two of weeks two and nine (see Figure 1). Warm-up was supervised by the club’s physical trainer, consisting of joint mobility and dynamic stretches, followed by low-intensity aerobic running and three progressive sprints performed at up to 95% of perceived effort. Players performed two 30 m sprints with a three-minute recovery between attempts. Verbal encouragement was given to ensure maximal voluntary effort. The average speed of the best attempt was recorded using photoelectric cells (Chronojump software, version 2.3.0-79, Barcelona, Spain).

##### Change of Direction (COD) 

The modified 505 test (505 m) [33] was conducted during the second session of weeks two and nine. One trial was performed to familiarize individuals with the execution dynamics. Two photoelectric barriers (Chronojump software, version 2.3.0-79, Barcelona, Spain) were placed, with a marker on the floor half a meter away from them. From this point, participants were instructed to run as quickly as possible for 5 m, turn 180°, and return to the start. Three attempts were performed, with participants changing direction with the right leg [34]. The best recorded time was used for analysis.

##### Optimal Load

Optimal load determination for sled dragging was conducted on the third day of the familiarization week (see Figure 1). To individualize the load magnitude, participants performed sprints with progressive loads (0%, 25%, 50%, and 75% of body mass) over 30 m for each attempt. The optimal load reduces speed by 50% compared to an unloaded sprint [35]. Calculations followed the procedure described by Romero-Franco et al. (2017) [36] using the MySprint^®^ application (version 2.1.0). Video recordings were made with an 8th generation iPad (Apple, Inc., Cupertino, CA, USA) placed perpendicular to the running surface, 10 m away, on a tripod at a height of 1.5 m. Six markers were placed at 5, 10, 15, 20, 25, and 30 m, with necessary parallelism corrections. Participants were encouraged to run as fast as the load allowed.

##### Training Protocol

**Figure 2:**
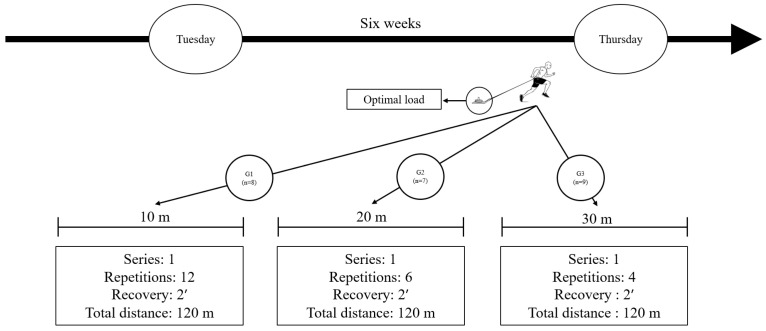
Design protocol resisted sprints, typical week. Duration: six weeks, weekly frequency: two sessions. G1: Experimental group 10 m; G2: Experimental group 20 m; G3: Experimental group 30 m.

Before each session, the team’s physical trainer conducted a standardized 10-min general warm-up. This included moderate-intensity linear running and changes of direction, bodyweight strength exercises such as squats and lunges, and dynamic flexibility drills. Players in the G10m, G20m, and G30m groups completed an equalized volume of 120 m of sled dragging with optimal load in each session. The repetition dosing was as follows: G10m: 12 repetitions, G20m: six repetitions, G30m, four repetitions. A two-minute recovery period was provided between attempts (see Figure 2).

##### Statistical Analysis

Data normality was assessed using the Shapiro-Wilk test, and variance homogeneity was checked using the Levene test. Differences were analyzed with a mixed ANOVA, incorporating a between-subjects factor (training group) and a within-subjects factor (pre- and post-intervention). Partial eta squared (η^2^p) was calculated to determine the effect sizes, providing a measure of the proportion of variance explained by each factor and their interaction. Effect sizes and percentage changes were also calculated. For effect sizes, Cohen’s d was used, with the following qualitative thresholds: trivial (<0.2), small (0.21–0.6), moderate (0.61–1.2), large (1.21–2), and very large (2.1–4) [37]. Percentage changes were calculated as described by Merino-Muñoz et al. (2020) [38]. Analyses were conducted using Microsoft Excel and IBM SPSS Statistics for Windows, version 29.0. Armonk, NY, USA: IBM Corp.

## 3. Results

### 3.1. Isometric Strength

The results for peak force (PF), time to peak force (TPF), impulse at 50 ms (I50), impulse at 100 ms (I100), and impulse at 200 ms (I200) are presented. All variables showed changes over time, irrespective of the training group (Table 1). No significant differences were found between groups when comparing pre- and post-intervention data (Table 1). However, differences were observed between groups for PF (*p* = 0.03; η^2^p = 0.26). Effect sizes for PF ranged from trivial to small, while TPF showed moderate effect sizes, and I50, I100, and I200 had small effect sizes. All groups demonstrated percentage changes associated with improved performance.

### 3.2. Vertical Jump

The results for jump height (JH), rate of force development (RFD), impulse at take-off (ID), yielding impulse (IY), braking impulse (IB), and concentric impulse (IC) are presented. All variables exhibited changes over time, regardless of the training group (Table 2). No significant differences were found between groups when comparing pre- and post-intervention data (Table 2). Similarly, there were no differences between groups. Effect sizes varied from trivial to moderate for JH, trivial to small for RFD, small to moderate for ID, trivial to moderate for IY, and trivial to small for IB and IC. All groups demonstrated percentage changes associated with improved performance, except for the 10 m group in IB (−0.84%) and the 20 m group in IC (−0.42%).

### 3.3. Change of Direction

Performance in the change of direction (COD) test showed improvements over time (*p* < 0.001, η^2^p = 0.46), regardless of the training group (*p* = 0.36, η^2^p = 0.10). No significant differences were found between groups when comparing pre- and post-intervention data (Table 3). Effect sizes ranged from small to moderate (ES = 0.44 to 0.90) across the groups. All groups demonstrated percentage changes associated with improved performance.

### 3.4. Linear Sprint

Average speed improved over time (*p* = 0.003, η^2^p = 0.36), regardless of the training group (*p* = 0.89, η^2^p = 0.01). No significant differences were observed between groups when comparing pre- and post-intervention data (*p* = 0.57, η^2^p = 0.05). Effect sizes ranged from small to moderate (ES = 0.37 to 0.62) across the groups (Table 4). All groups showed percentage changes indicative of performance improvement.

## 4. Discussion

Resisted sprint training has emerged as an effective method for enhancing the acceleration phase of sprinting [14,15]. Previous studies have reported improvements in soccer-specific actions such as linear sprints [39], change of direction (COD) [10], and accelerations [24]. Despite the general agreement on the benefits of this training method, the influence of sprint distance on adaptive responses remains to be determined. This study aimed to analyze the impact of different resisted sprint distances on muscular performance in young soccer players. It was hypothesized that distance would significantly influence muscular adaptation in high-intensity efforts. The results indicate that adaptations occurred regardless of the resisted sprint distance, albeit with varying magnitudes.

Our findings indicate an improvement in change of direction (COD) performance for all sprint distances. These results are consistent with previous research. Gil et al. (2018) [11] implemented a resisted and unloaded sprint training protocol with adult soccer players over six weeks, using a load that reduced maximum speed by 10%. They observed a 6.1% improvement in COD performance time, although there were no significant differences compared to the unloaded group. Their protocol also included overloaded jumps (60% BM), which makes it difficult to attribute the improvements solely to resisted training. Additionally, they used a device that did not allow for sprints at distances suitable for the development of acceleration and maximum speed (7 m linear sprint), limiting the ability to evaluate the influence of sprint distance on performance. In our case, the strength stimulus was limited exclusively to sled drags, with no other specific strength exercises included, and only the technical-tactical sessions corresponding to the planned training for those days were added. Similarly, Pareja-Blanco et al. (2019) [40] designed a protocol with five training groups, based on resisted sprints with high and low loads (LST—12.5% BM and HST—80% BM, respectively), as well as resisted sprints with high and low loads combined with vertical jumps with overload (LST + SQ and HST + SQ, respectively), and a group performing jumps with overload (SQ). Their findings showed that only LST + SQ, HST + SQ, and LST improved COD performance. Consistent with our results, improvements in COD were observed without the need to add an extra stimulus, with the caveat that the sled load was on average higher than the one described in Gil et al.’s study. A possible explanation for this phenomenon could be found in the lower level of expertise of the study subjects, which increases the responsiveness regardless of the magnitude of the stimulus.

Recently, Loturco et al. (2024) [27] have questioned the effectiveness of resisted sprints as a strategy to optimize high-intensity actions in young soccer players. In their study, two training protocols were designed: one based on squat jumps and another on resisted runs with a load equivalent to 30% of body mass. The results indicated significant improvements in vertical jump height only in the group that performed squat jumps, while the resisted running group showed no relevant progress in the performance variables assessed. These findings led the authors to suggest that the inclusion of resisted sprints at the beginning of the season may be questionable due to the lack of positive effects on sprint and jump performance.

One of the key observations by Loturco et al. (2024) [27] concerns the timing of the season when these training strategies are implemented. Previous studies have reported performance improvements using resisted sprints, but these interventions were conducted during the competitive period, suggesting a possible interaction with the specific demands of that stage. In line with this background, our findings show that applying the training protocol during the competitive period could explain the observed increases in change of direction (COD) performance. Therefore, it is plausible that the improvements obtained are influenced by the synergy between the implemented protocol and the demands of competition.

In this regard, Mainer-Pardos et al. (2024) [28] point out that although resisted sprint training contributes to improving COD performance, its effects are enhanced when combined with stimuli performed in the vertical plane. However, it is not possible to attribute the effectiveness of this strategy exclusively to resisted sprints, as the studies reviewed did not include comparisons with a non-resisted control group; instead, only within-group comparisons were conducted. Regarding our findings, they could be explained by the level of expertise of the players included in the sample. Since the participants had limited prior exposure to this type of training and, therefore, had a greater adaptation reserve, a relatively low training dose was sufficient to generate improvements in their performance.

The impact of resisted sprint training on linear sprint performance has been well-documented in the literature [16,21,28]. However, the influence of sprint distance on these adaptations still needs to be conclusive. Rodríguez-Rosell et al. (2022) [41] implemented a resisted sprint protocol with five different load magnitudes, ranging from 0 to 80% BM, over a fixed distance of 20 m. All groups showed speed improvements, ranging from 0.8% for 80% BM to 1.5% for 40% BM. Similarly, Bachero-Mena & González-Badillo (2014) [20] conducted 14 sessions of resisted sprints with differential loads (5 [LL]–12.5 [ML]–20 [HL] % BM) over distances between 20 and 35 m over seven weeks with physically active students. Results demonstrated improved times over 30 m for all groups, with significant improvements (*p* < 0.001) observed in the HL group. The authors noted that high loads primarily affect the initial meters of the sprint, specifically during the acceleration phase. Our findings support this assertion, as the G20 group experienced the most significant improvement (ES = 0.62; Δ 3.2%). Comparable results were reported by West et al. (2013) [42], who conducted 12 sessions of resisted sprints (12.5% BM) with professional rugby players (n = 20). The largest effect sizes for performance over 10 and 30 m were observed in the resisted sprint group compared to the unloaded sprint group, with a sprint distance of 20 m, consistent with our findings.

Our findings on the effects of resisted sprints on vertical jump kinematics indicate improvements in force production per unit of time and vertical jump height. Previous research supports these results, with similar protocols showing enhanced performance in these variables. For instance, Sinclair et al. (2021) [43] demonstrated significant improvements in vertical jump height following 16 sessions over eight weeks of resisted sprint training with professional rugby players. Specifically, 20-m sprints with a load of 25.0–26.9% BM and a total volume of 180 m per session resulted in a 6.5% increase in vertical jump height (from 40.43 ± 3.87 cm to 43.07 ± 4.55 cm). Conversely, results for metrics derived from the force-time curve are less consistent. Harrison & Bourke (2009) [44] conducted 12 sessions of resisted sprints over 20 m (~13% BM, 120 m per session), analyzing possible effects on force-time relationships through vertical jumps without countermovement (SJ). Their results showed no improvements in the rate of force development (RFD) or the time to reach the maximum rate of force development (*p* values of 0.502 and 0.296 for time; 0.738 and 0.245 for time x group, respectively). The absence of a stretch-shortening cycle in the selected jump gesture may have limited the expression of the adaptation. This could explain the lack of improvements in force production per unit of time. However, this assertion might be challenged by the results of Alcaraz et al. (2012) [45], who also found no improvements in RFD for countermovement jumps.

Kinetic variables derived from the isometric mid-thigh pull (IMTP) test also showed improvements across all three experimental groups. According to our literature review, there is a lack of evidence regarding the effects of resisted sprint training on these metrics. However, peak force (PF) measured through static strength tests has demonstrated moderate to high correlations with various performance expressions. Comfort et al. (2019) [30] reported moderate to high correlations between PF, as assessed by the IMTP, and performance in change of direction (COD), 20-m sprints, and countermovement jumps (r = −0.57 to 0.79, *p* < 0.05; r = −0.69, *p* < 0.05; r = 0.59 to 0.82, *p* < 0.05, respectively). Given the observed improvements in these performance variables, it is plausible to infer that the increases in PF observed across all three groups could be attributed to these overall enhancements in muscular performance.

To our knowledge, no original studies have specifically examined the influence of sprint distance on physical performance in any population. From this perspective, our study is pioneering in its approach, as it evaluates different sprint distances with homogenized volume and individualized load within the same design. Rumpf et al. (2016) [46] reviewed the effects of various speed training methods on sprint performance over different distances. Their conclusions highlighted that resisted sprint training was most effective for improving performance over distances of 20 m, with greater effectiveness observed as the drag load increased (over 10% body mass or 10% velocity decrement). An important aspect to consider is the influence of load magnitude on running mechanics. In this regard, Zabaloy et al. (2023) [9] warn that loads causing a velocity loss greater than 30% negatively impact running technique, effectively transforming the sprint into a heavy-loaded march. From this perspective, such a method could be considered a tertiary approach to sprint development, akin to strength training with loads close to 1RM. This aspect should be taken into account by physical conditioning coaches.

One limitation of our study is that we did not control for split times during the 30-m sprint. This omission prevents us from providing insights into how sprint distance might influence the acceleration phase of sprinting. Similarly, the limited experience of the selected sample in high-intensity strength training may have contributed to the observed adaptations. The literature strongly emphasizes this point, particularly the inverse relationship between the magnitude of changes and the athletes’ level of expertise. Finally, the inclusion of internal load markers (e.g., lactate, heart rate, muscle soreness) could contribute to the better understanding of adaptive responses in young soccer players [47,48,49].

Overall, our study strengthens the body of evidence supporting the benefits of specific training strategies for enhancing performance in team sports. In particular, our design introduces new elements that had not been previously explored: (i) Individualization of the load based on optimal/high load parameters in developing soccer players, (ii) Comparison of different sprint distances within the same design, and (iii) Examination of the effects of resisted sprints on metrics of the force-time curve in both static and dynamic tests.

## 5. Conclusions

Our findings have significant implications for coaches and trainers working with young soccer players. We observed performance improvements in vertical jump, isometric strength, sprinting, and change of direction, regardless of the sprint distance used in resisted sprint training. No significant differences were found based on the sprint distance. Therefore, our study suggests that sprint distances ranging from 10 to 30 m, with individualized loads, could be equally effective for enhancing muscular performance in young soccer players.

## Figures and Tables

**Table 1:** Pre- and post-test scores (Mean [SD]), Effect Size, and Percentage of Change in Isometric Mid-thigh Pull Performance.

		Pre		Post		ES	PC (%)	Time		Time and Group		Group	
	Group	Mean	SD	Mean	SD			*p*	η^2^p	*p*	η^2^p	*p*	η^2^p
PF (N)	10 m	1660	310	1713	325	0.17	3.19	<0.001	0.62	0.30	0.12	0.03	0.26
	20 m	1441	185	1472	177	0.18	2.20						
	30 m	1836	121	1888	133	0.42	2.89						
TPF (ms)	10 m	2.85	1.32	1.77	0.84	0.97	−37.75	<0.001	0.53	0.81	0.03	0.48	0.08
	20 m	2.49	1.17	1.68	1.37	0.64	−32.53						
	30 m	2.06	1.13	1.38	1.07	0.62	−32.83						
Impulse 50 ms (N·kg)	10 m	40.81	8.77	43.8	11.2	0.30	7.44	<0.001	0.57	0.58	0.07	0.17	0.16
	20 m	34.67	5.51	36.2	5.80	0.27	4.44						
	30 m	42.25	5.14	44.6	5.98	0.42	5.49						
Impulse 100 ms (N·kg)	10 m	89.32	20.30	98.1	27.3	0.37	9.87	<0.001	0.60	0.30	0.12	0.15	0.16
	20 m	74.74	11.70	78.9	12.1	0.35	5.60						
	30 m	92.87	13.38	99.7	15.1	0.47	7.29						
Impulse 200 ms (N·kg)	10 m	209	50.38	227.5	61.5	0.34	9.19	<0.001	0.67	0.21	0.14	0.11	0.19
	20 m	171	27.23	182	25.3	0.40	6.15						
	30 m	217	35.80	235	36.9	0.49	8.17						

PF: Peak force; TPF: Time of peak force; ES: Effect size; PC: Percent of change.

**Table 2:** Pre- and post-test scores (Mean [SD]), Effect Size, and Percentage of Change in Vertical Jump Performance.

		Pre		Post		ES	PC (%)	Time		Time and Group		Group	
	Group	Mean	SD	Mean	SD			*p*	η^2^p	*p*	η^2^p	*p*	η^2^p
JH (m)	10 m	0.33	0.03	0.36	0.05	0.69	9.09	<0.001	0.64	0.17	0.16	0.92	0.01
	20 m	0.32	0.05	0.33	0.06	0.2	3.13						
	30 m	0.33	0.05	0.35	0.06	0.41	6.06						
RPD (W/s)	10 m	307	104.8	312	93.6	0.04	1.38	0.041	0.137	0.659	0.053	0.292	0.119
	20 m	292	94.2	324	131.7	0.28	11.02						
	30 m	248	41.9	266	32.4	0.48	7.27						
ID (N·kg)	10 m	39.4	11.7	43.4	13.6	0.31	10.16	0.003	0.27	0.791	0.035	0.253	0.129
	20 m	32.1	5.4	39.3	11.0	0.82	22.22						
	30 m	42.0	8.2	48.4	10.7	0.67	15.34						
IY (N·kg)	10 m	36.4	10.8	48.3	12.6	1.01	32.65	<0.001	0.492	0.009	0.327	0.184	0.151
	20 m	39.3	12.3	40.0	10.7	0.06	1.82						
	30 m	45.7	8.4	50.3	10.7	0.48	10.22						
IB (N·kg)	10 m	74.3	18.1	73.6	13.7	0.04	−0.84	0.235	0.048	0.792	0.035	0.067	0.215
	20 m	57.9	12.8	63.9	26.7	0.29	10.37						
	30 m	79.3	21.6	89.9	16.7	0.55	13.31						
IC (N·kg)	10 m	155	23.9	161	21.6	0.24	3.46	0.014	0.19	0.443	0.087	0.04	0.246
	20 m	135	17.1	134	16.5	0.03	−0.42						
	30 m	160	13.9	165	12.5	0.39	3.19						

JH: Jump height; RPD: Rate power development; ID: Impulse discharge; IY: Yielding impulse; IB: Braking impulse; IC: Concentric impulse; ES: Effect size; PC: Percent of change.

**Table 3:** Pre- and post-test scores (Mean [SD]), Effect Size, and Percentage of Change in COD Performance.

		Pre		Post		ES	PC (%)	Time		Time and Group		Group	
	Group	Mean	SD	Mean	SD			*p*	η^2^p	*p*	η^2^p	*p*	η^2^p
Time (s)	10 m	2.58	0.12	2.47	0.16	0.75	−4.16	<0.001	0.46	0.36	0.1	0.54	0.07
	20 m	2.54	0.10	2.41	0.17	0.9	−5.00						
	30 m	2.57	0.11	2.52	0.10	0.44	−1.84						

ES: Effect size; PC: Percent of change.

**Table 4:** Pre- and post-test scores (Mean [SD]), Effect Size, and Percentage of Change in Sprint Performance.

		Pre		Post		ES	PC (%)	Time		Time and Group		Group	
	Group	Mean	SD	Mean	SD			*p*	η^2^p	*p*	η^2^p	*p*	η^2^p
Mean Speed (m/s)	10 m	6.07	0.27	6.17	0.24	0.37	1.61	0.003	0.36	0.89	0.01	0.57	0.05
	20 m	6.08	0.23	6.22	0.21	0.62	2.30						
	30 m	5.97	0.15	6.10	0.25	0.58	2.08						

ES: Effect size; PC: Percent of change.

## Data Availability

Data are available for research purposes upon request to the corresponding author.
